# Nucleus-Cytoskeleton Crosstalk During Mitotic Entry

**DOI:** 10.3389/fcell.2021.649899

**Published:** 2021-03-18

**Authors:** Margarida Dantas, Joana T. Lima, Jorge G. Ferreira

**Affiliations:** ^1^Instituto de Investigação e Inovação em Saúde – i3S, University of Porto, Porto, Portugal; ^2^BiotechHealth Ph.D. Programme, University of Porto, Porto, Portugal; ^3^Instituto de Ciências Biomédicas Abel Salazar (ICBAS), University of Porto, Porto, Portugal; ^4^Departamento de Biomedicina, Faculdade de Medicina, University of Porto, Porto, Portugal

**Keywords:** mitosis, nucleus, cytoskeleton, centrosome, mechanotransduction, chromosome, nuclear lamina

## Abstract

In preparation for mitosis, cells undergo extensive reorganization of the cytoskeleton and nucleus, so that chromosomes can be efficiently segregated into two daughter cells. Coordination of these cytoskeletal and nuclear events occurs through biochemical regulatory pathways, orchestrated by Cyclin-CDK activity. However, recent studies provide evidence that physical forces are also involved in the early steps of spindle assembly. Here, we will review how the crosstalk of physical forces and biochemical signals coordinates nuclear and cytoplasmic events during the G2-M transition, to ensure efficient spindle assembly and faithful chromosome segregation.

## Introduction

**FIGURE 1:**
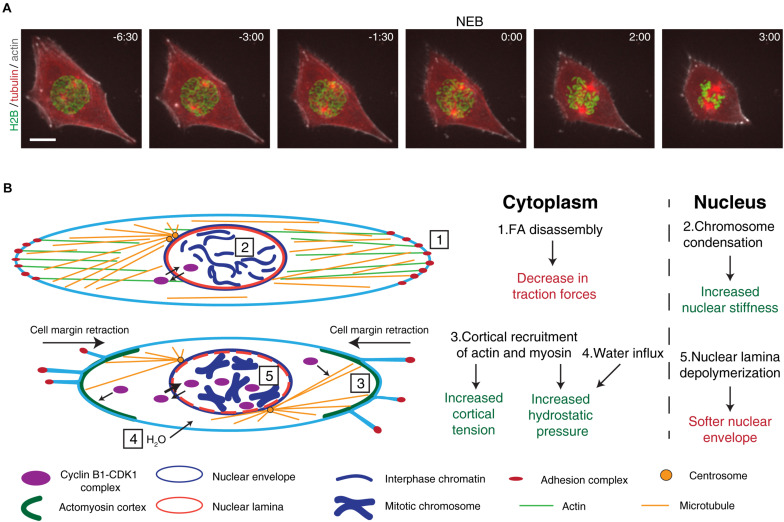
Overview of the cytoskeletal and nuclear reorganization that occur during mitotic entry. **(A)** Representative frames from a movie of a RPE-1 cell expressing H2B-GFP/tubulin-RFP/SiR-actin during mitotic entry. It is possible to observe the main events that occur during mitotic entry, such as cell rounding, chromosome condensation, and centrosome separation. After NEB, mitotic rounding continues as the spindle assembles. Time is in min:sec. Scale bar, 10 μm. Time zero corresponds to NEB. **(B)** Main events that occur during the G2-M transition. Cyclin B1-CDK1 complexes shuttle between the cytoplasm and the nucleus. At this stage, the cell is attached to the extracellular matrix through membrane-bound adhesion complexes (1) and the microtubule and actin cytoskeletons are in their interphase configuration. Inside the nucleus, chromatin is decondensed and the nuclear envelope and nuclear lamina are intact (2). As cells prepare to enter mitosis, adhesion complexes disassemble, leading to cell membrane retraction and mitotic cortex assembly (3). Together with osmotic swelling (4), this leads to increased intracellular pressure. At the same time, active cyclin B1-CDK1 complexes accumulate in the nucleus, triggering chromosome condensation, nuclear lamina depolymerization (5), and nuclear envelope permeabilization. These events trigger changes global changes in the forces during the G2-M transition.

An efficient mitosis is required to maintain genomic stability and ensure correct tissue development and homeostasis. While nuclear envelope breakdown (NEB) marks the irreversible step of mitotic commitment, the process starts well before, as chromosomes condense (Antonin and Neumann, 2016) and centrosomes separate (Whitehead et al., 1996). This occurs simultaneously with a global reorganization of the microtubule and actin cytoskeletons. Accordingly, the interphase microtubule cytoskeleton disassembles (Mchedlishvili et al., 2018) and overall microtubule dynamics change (Zhai et al., 1996), which allows the formation of a bipolar spindle (Heald and Khodjakov, 2015) required for accurate chromosome capture (Figure 1). At the same time, the interphase actin cytoskeleton is replaced with a mitotic actomyosin network that is connected with the plasma membrane (Chugh and Paluch, 2018) and drives mitotic rounding (Rosa et al., 2015). Importantly, timely progression through these steps requires the activity of mitotic kinases such as CDK1 and PLK1 (Gavet and Pines, 2010b; Ramanathan et al., 2015; Gheghiani et al., 2017). Simultaneously, within the nucleus, a cascade of events regulated by the same mitotic kinases initiate chromosome condensation (Abe et al., 2011) and trigger disassembly of the nuclear pore complex (NPC; Linder et al., 2017) and nuclear lamina (NL; Heald and McKeon, 1990; Peter et al., 1990).

Here, we will discuss how the interactions between the cytoskeleton and nucleus set the stage for spindle assembly and how the prophase nucleus acts as more than a passive player to ensure a successful mitosis.

## Mitotic Cell Rounding

Mitotic cell rounding is a feature of a large number of eukaryotic cells that lack a cell wall (Mitchison, 1992; Gibson et al., 2006; Thery and Bornens, 2008). However, this is not a universal characteristic, as some metazoan cells such as Ptk1 or newt pneumocytes are still capable of progressing through mitosis without rounding (Roos, 1973; Hayden et al., 1990; Rieder and Alexander, 1990). The rounding process is regulated by CDK1 activity (Jones et al., 2018) and starts in the early stages of mitosis (Matthews et al., 2012) with the loss of Arp2/3-dependent lamellipodia (Bovellan et al., 2014) and disassembly of focal adhesions (FAs; Dao et al., 2009). This loss of FAs leads to the decrease in cell traction forces observed during G2 (Uroz et al., 2018; Vianay et al., 2018) and prophase (Nunes et al., 2020) and allows cell margin retraction (Mitchison, 1992; Maddox and Burridge, 2003) (Figure 1). In turn, this change in cell shape enables the formation of a stiff actomyosin cortex (Maddox and Burridge, 2003; Kunda et al., 2008; Fischer-Friedrich et al., 2016), through the CDK1-mediated phosphorylation of Myosin II (Ramanathan et al., 2015) and Ect2, a RhoGEF that activates the RhoA GTPase (Matthews et al., 2012). In combination with an increase in hydrostatic pressure (Stewart et al., 2011) and cell volume (Zlotek-Zlotkiewicz et al., 2015), likely driven by water influx (Son et al., 2015), these changes provide the necessary space for mitotic spindle assembly and accurate chromosome capture (Kunda et al., 2008; Lancaster et al., 2013). Consequently, a failure in mitotic cell rounding triggered by either blocking FA disassembly or mechanical compression leads to defects in spindle assembly and mitotic progression (Lancaster et al., 2013; Nunes et al., 2020) and increases chromosome missegregation (Tse et al., 2012; Lancaster et al., 2013; Cattin et al., 2015; Matthews et al., 2020). The need for cell rounding was further emphasized with the proposal of an “adhesion-dependent checkpoint,” which acts through DEPDC1B to inhibit RhoA activation and allow FA dismantling during the G2-M transition (Marchesi et al., 2014), required for normal proliferation and development of zebrafish embryos.

## Centrosome Separation and Spindle Assembly

In animal cells, spindle assembly originates mainly from the centrosomes. For this reason, many studies have focused on centrosome behavior during the early stages of mitosis. Initial centrosome separation requires the combined action of microtubule-associated molecular motors such as kinesin-5 and dynein (for review, see Tanenbaum and Medema, 2010). The plus-end directed kinesin-5 has a homo-tetrameric structure that can crosslink and slide anti-parallel microtubules apart (Kashina et al., 1996). This generates pushing forces on microtubules that lead to centrosome separation (Whitehead et al., 1996). For this reason, kinesin-5 has been involved in spindle assembly in nearly all model systems analyzed (Sawin et al., 1992; Heck et al., 1993; Blangy et al., 1995), with the exception of *C. elegans* (Bishop et al., 2005). Dynein, on the other hand is a microtubule minus-end directed motor (Roberts et al., 2013). To generate the pulling forces necessary for centrosome separation, dynein needs to be tethered to sub-cellular structures such as the nuclear envelope (NE; Splinter et al., 2010; Bolhy et al., 2011; Nunes et al., 2020) or the cell cortex (Kotak et al., 2012). The combined activity of these motors is sufficient to drive centrosome separation, but it does not explain the biased movement of centrosomes to the shortest axis of the nucleus (Magidson et al., 2011; Nunes et al., 2020). Such a bias would require additional cues (either external or internal) or an asymmetry in the forces exerted on the centrosomes, to direct centrosome movement. Notwithstanding, the extent of centrosome separation, as well as their positioning at the moment of NEB, remain major contributors to chromosome missegregation events. Failure to fully separate centrosomes during mitotic entry can contribute to deviant spindle morphologies (Silkworth et al., 2012; Nam et al., 2015), increasing the likelihood of generating erroneous kinetochore-microtubule attachments. Most of these attachments are sensed by the Spindle Assembly Checkpoint (SAC), which generates a “wait-anaphase” signal until all chromosomes are correctly attached (Lara-Gonzalez et al., 2012). However, merotelic attachments, which occur when one kinetochore is bound to microtubules emanating from different poles, are usually invisible to the SAC (Gregan et al., 2011). Consequently, cells with incompletely separated centrosomes at NEB tend to have a higher rate of chromosome missegregation (Kaseda et al., 2012; Silkworth et al., 2012; Nunes et al., 2020).

During metaphase, cortical force generators dictate spindle orientation (Thery et al., 2007; Kotak et al., 2012) by sensing external cues (Thery et al., 2005; Toyoshima and Nishida, 2007; Fink et al., 2011). However, during the initial stages of mitosis, as cells round up and the actomyosin cortex is yet to be assembled, these cortical force generators are not present (Kiyomitsu and Cheeseman, 2012; Kotak et al., 2012). Therefore, it is likely that the cues required for centrosome positioning during early mitosis are not provided by external signals, but rather derive from an internal input. One such signal could be provided by the NE-specific pool of dynein, that is dependent on association with the RanBP2-BicD2 (Splinter et al., 2010) or Nup133/CENP-F/NudE-NudEL (Bolhy et al., 2011) pathways, in a CDK1-dependent manner (Baffet et al., 2015). Accordingly, preventing dynein loading on the NE results in a failure to separate (van Heesbeen et al., 2013; De Simone et al., 2016; Boudreau et al., 2019) and correctly position centrosomes (Splinter et al., 2010; Bolhy et al., 2011; Nunes et al., 2020). The manner in which the properties of the prophase nucleus dictate dynein localization and activity to ensure positioning of centrosomes on the shortest nuclear axis and avert chromosome missegregation remains an open question.

## The Nucleus and Nucleo-Cytoskeletal Coupling

The cell nucleus is encased by a NE that acts as a barrier between cytoplasmic and nuclear components. The NE is composed of and inner (INM) and an outer (ONM) nuclear membrane, NPCs and a dense NL. The NL consists mainly of A-type and B-type Lamins, which are type V intermediate filaments that provide structural support to the nucleus (Dechat et al., 2010). Lamins can interact with chromatin and with NE membrane proteins, such as Emerin, LAP2, or nuclear soluble factors such as barrier-to-autointegration factor (BAF) (Ungricht and Kutay, 2017).

The nucleus is continuously under the influence of external forces. When physical forces are applied to the cell, they are decoded into biochemical signals in a process known as mechanotransduction. This process starts at the cell membrane, where adhesion complexes sense external cues (Sun et al., 2016). The cytoskeleton then relays these signals to the nucleus through the linker of nucleoskeleton and cytoskeleton (LINC) complex (Lombardi and Lammerding, 2011), which triggers a nuclear mechanical response that depends on the NL (Stephens et al., 2017), chromatin condensation (Schreiner et al., 2015; Stephens et al., 2017) and nucleo-cytoskeletal coupling (Lombardi and Lammerding, 2011). This ultimately leads to changes in nuclear structure and organization (Lammerding, 2011; Maurer and Lammerding, 2019) and regulates cell cycle progression (Uroz et al., 2018; Vitiello et al., 2019).

As mentioned above, a series of well-coordinated events ensure timely mitotic entry, starting with chromosome condensation (Antonin and Neumann, 2016) and cytoskeletal reorganization (Ramkumar and Baum, 2016; Champion et al., 2017), and culminating in nuclear permeabilization (Beaudouin et al., 2002; Salina et al., 2002). In higher eukaryotes, nuclear permeabilization starts with the removal of nucleoporins from NPCs (Dultz et al., 2008; Katsani et al., 2008), which triggers a loss of the nucleo-cytoplasmic boundary. The process continues with the contribution of dynein-driven, microtubule-dependent pulling forces, which generate holes in the nucleus and assist in membrane clearing from chromosomes (Beaudouin et al., 2002; Salina et al., 2002; Muhlhausser and Kutay, 2007). Finally, the NL depolymerizes, due to Lamin phosphorylation and consequent nucleoplasmic release (Heald and McKeon, 1990; Peter et al., 1990; Georgatos et al., 1997). These steps are essential to allow the interaction of microtubules with kinetochores on mitotic chromosomes. In interphase, the mechanical response of the nucleus is dictated by the chromatin condensation state (Stephens et al., 2017), the levels of Lamin A (Buxboim et al., 2017) and the interaction of heterochromatin with the nuclear membrane (Schreiner et al., 2015). Remarkably, as cells transition from G2 to mitosis, all the above components are extensively modified. Phosphorylation of Lamin A by CDK1 (Heald and McKeon, 1990; Peter et al., 1990), triggers its disassembly from the NL and consequent release into the nucleoplasm (Georgatos et al., 1997). Although direct measurements of nuclear stiffness at this stage have not been made, it is possible to assume that NL depolymerization significantly changes the mechanical response of the nucleus, facilitating NEB. Accordingly, MEFs with Lamin A/C deficiency show impaired nuclear stiffness and mechanics (Lammerding et al., 2004, 2006). This is in line with observations in human cells, showing that loss of Lamin A renders nuclei softer (Pajerowski et al., 2007) and prone to rupture (Earle et al., 2020). Taken together, these observations implicate the NL in the mechanical stability of the nucleus and highlight the need for its depolymerization during prophase (Georgatos et al., 1997), to facilitate microtubule-dependent nuclear permeabilization (Beaudouin et al., 2002; Salina et al., 2002). At the same time, mitotic chromosomes condense, altering their structure and stiffness (Stephens et al., 2017; Sun et al., 2018; Biggs et al., 2019). Evidence from metaphase chromosomes isolated from HeLa cells showed this process to be largely dependent on condensins (Sun et al., 2018), although histone post-translational modifications also play an important role (Biggs et al., 2019). Finally, the actin cytoskeleton, which is connected to the nucleus through the LINC complex (Versaevel et al., 2014), is remodeled to assemble a mitotic cortex (Ramkumar and Baum, 2016). This remodeling might modify the connections between the cytoskeleton and the nucleus, contributing to changes in nuclear mechanics. Accordingly, disrupting the actin cytoskeleton in NIH3T3 cells was sufficient to modify the compressive forces exerted on the nucleus and induce changes in chromatin organization (Li et al., 2014). Taken together, these studies suggest that the mechanical properties of the nucleus change during the G2-M transition and warrant further investigation on the functional relevance of nuclear mechanics for mitotic fidelity.

**FIGURE 2:**
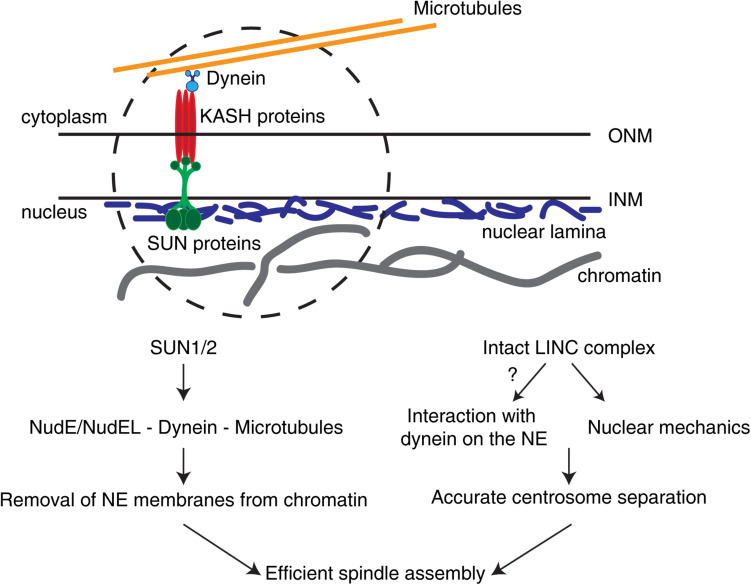
The LINC complex in early spindle assembly and chromosome segregation. The LINC complex consists of SUN1/2 trimers on the inner nuclear membrane (INM) and KASH proteins on the outer nuclear membrane (ONM). In somatic cells, different KASH proteins differentially bind to specific motors (e.g., dynein) or to distinct cytoskeletal components. These complexes are able to sense forces relayed by the cytoskeleton and transmit them to the nuclear interior. During the G2-M transition, SUN proteins are required to remove NE membranes from chromatin and position centrosomes. In addition, an intact LINC complex is necessary for correct centrosome separation. Whether this is due to LINC complex-dependent loading of dynein on the NE or to nuclear mechanotransduction remains unclear.

While measurements of the mechanical properties of the nucleus during the G2-M transition are still missing, there is already significant evidence to support a role for the nucleus and nucleus-associated components in other steps of mitosis, namely in determining chromosome segregation fidelity. One key component in nuclear mechanotransduction is the aforementioned LINC complex (Figure 2). This complex consists of SUN (Sad1, UNC84) proteins in the INM and KASH (Klarsicht, ANC-1, and Syne Homology)-containing proteins in the ONM (Starr and Fridolfsson, 2010). Importantly, studies in MEFs using a microneedle assay to apply controlled cytoskeletal strains, in combination with dominant-negative forms of SUN and KASH proteins, showed that an intact LINC complex is essential for force transmission to the nucleus (Lombardi et al., 2011). Similarly, in cultured human cells, depletion of both SUN1 and SUN2 delayed NE disassembly (Figure 2), similarly to what is observed after microtubule depolymerization with nocodazole (Turgay et al., 2014). Consequently, centrosome separation is disrupted (Stiff et al., 2020) and mitotic progression affected (Turgay et al., 2014). Moreover, an intact LINC complex is essential during early mitosis for decreasing chromosome scattering (Booth et al., 2019), likely facilitating their capture and congression (Booth et al., 2019; Stiff et al., 2020). Importantly, the LINC complex also directly associates with dynein on the NE to control nuclear migration (Malone et al., 2003; Zhang et al., 2009; Fridolfsson and Starr, 2010; Yu et al., 2011) and meiotic chromosome movement (Chikashige et al., 2006; Sato et al., 2009). Given that an intact LINC complex is required for force transmission to the nucleus (Lombardi et al., 2011) and NE dynein is essential for centrosome positioning (Nunes et al., 2020), it is possible that LINC-mediated mechanical forces could play an important part in determining correct centrosome positioning by ensuring timely dynein loading. Accordingly, depletion of SUN1 and SUN2 is sufficient to abolish NE dynein localization (Turgay et al., 2014; Nunes et al., 2019). Whether this is directly due to a defect in nuclear mechanotransduction triggered by loss of the LINC complex remains unknown (Figure 2).

Other nuclear components have also been implicated in spindle assembly and chromosome segregation. Blocking the removal of NE membranes at mitotic onset leads to defects in spindle assembly and chromosome segregation (Turgay et al., 2014; Champion et al., 2019). Similar defects in membranes removal could also be triggered by expression of a mutant version of Lamin A that is observed in progeria patients (Dechat et al., 2007). However, Lamin A, together with BAF and LAP2α, is also directly involved in spindle assembly and orientation by targeting dynein to the cell cortex (Qi et al., 2015). Moreover, chromosome distribution is altered in LMNA mutant fibroblasts (Meaburn et al., 2007). Such alterations could directly affect chromosome distribution during early mitosis, disrupting the disk-like prometaphase chromosome organization, essential for spindle assembly (Magidson et al., 2011). Taken together, these defects could explain why Lamin A/C deficiency leads to aneuploidy and chromosomal instability (Dechat et al., 2007; Capo-chichi et al., 2011; Capo-Chichi et al., 2016; Smith et al., 2018). Interestingly, mitotic problems are not exclusive to Lamin A. In *C. elegans*, it was shown that reduced levels of MAN1 and Emerin, INM proteins which interact with Lamins and the LINC complex (Piccus and Brayson, 2020), trigger “anaphase-bridged chromatin” (Liu et al., 2003), a phenotype also observed in a mouse model of laminopathy (Pratt et al., 2011), and in human cells with reduced Lamin A levels (Cao et al., 2007). Moreover, loss of Lamin B2 in human cells was also shown to trigger chromosomal instability, by interfering with the spatial organization of chromosomes (Ranade et al., 2017) and affecting spindle assembly (Kuga et al., 2014).

Although these reports are compelling, there are alternative hypotheses to explain how alterations in Lamins could indirectly trigger mitotic defects. Chromatin is thought to associate with the NL through specific sequences known as lamina-associated domains (LADs) (van Steensel and Belmont, 2017) that are considered to be transcriptionally repressive regions (Guelen et al., 2008) and help organize chromosomes within the nuclear volume (Mewborn et al., 2010). Notably, Lamin A phosphorylation on Ser22, essential for NL depolymerization during mitotic entry (Heald and McKeon, 1990), was recently shown to act as a transcriptional regulator (Ikegami et al., 2020), which could explain why LMNA mutants show altered gene expression patterns (Mewborn et al., 2010). Whether the mitotic defects triggered by Lamin A loss could be due to changes in its transcriptional program remains to be determined.

## Mechanical Forces in Cell Cycle Progression

The link between mechanical forces and the cell cycle has long been recognized (Chen et al., 1997; Huang et al., 1998). In capillary endothelial cells, tractional forces are sufficient to trigger the G1-S transition by increasing Cyclin D1 levels and down-regulating the cell cycle inhibitor p27^Kip^ (Huang et al., 1998). This likely occurs by force-mediated nuclear deformation that triggers the activation of transcription factors such as TEAD and AP1, leading to the induction of genes that promote the G1-S transition (Aureille et al., 2019). In agreement with these observations, recent data obtained in MDCK monolayers showed that both tension and mechanical energy are good predictors of G1 duration (Uroz et al., 2018).

Other stages of the cell cycle are also mechanically regulated. In fact, the organization pattern of actomyosin forces sets the duration of the S and G2 phases, by modulating centriole duplication and Plk4 recruitment (Vitiello et al., 2019). In addition, there is evidence from MDCK monolayers and isolated cells, for a decrease in cell traction forces during G2 and early mitosis (Uroz et al., 2018; Vianay et al., 2018; Nunes et al., 2020), which occurs in tandem with the disassembly of FAs (Dao et al., 2009) and an increased expression of DEPDC1B (Marchesi et al., 2014). How these events are coordinated is still unclear. It is possible that, during the G2-M transition, a FA-generated mechanical signal is relayed from the cell membrane to the nucleus, triggering DEPDC1B expression, which would then act as a RhoA inhibitor to regulate adhesion dynamics (Marchesi et al., 2014). This, together with increased CDK1 activity (Jones et al., 2018), would set the timing for FA disassembly and mitotic entry (Gavet and Pines, 2010a,b; Marchesi et al., 2014).

## Conclusion

Efficient assembly of a mitotic spindle requires accurate coordination between cytoplasmic and nuclear events. This is achieved, at least partly, by the activity and localization of the Cyclin B1-CDK1 complex (Gavet and Pines, 2010a,b). In the cytoplasm, CDK1 enables centrosome separation (Smith et al., 2011) and induces global changes in microtubule dynamics by directly phosphorylating microtubule-associated proteins (MAPs) and modifying their microtubule binding capacity (Lamb et al., 1990; Verde et al., 1990; Verde et al., 1992). On the other hand, inside the nucleus, CDK1 contributes to NPC disassembly (Linder et al., 2017) and NL depolymerization (Heald and McKeon, 1990; Peter et al., 1990). These biochemical events trigger a global cellular reorganization that allows the assembly of an actomyosin cortex and a microtubule-based mitotic spindle.

In addition to the biochemical pathways controlling mitotic entry, it has long been proposed that mechanical forces also regulate the cell cycle (Huang et al., 1998; Lancaster et al., 2013; Uroz et al., 2018; Vianay et al., 2018; Aureille et al., 2019). High cellular tension triggers a transition from G1 to S phase (Huang et al., 1998; Uroz et al., 2018; Aureille et al., 2019) and also regulates the length of the S-G2 phases of the cell cycle (Vitiello et al., 2019). In part, this could be due to tension-generated NE deformation that is sufficient to trigger mechanically-activated transcriptional programs (Aureille et al., 2019) and affect cell proliferation (Versaevel et al., 2012). As cells progress toward mitosis, tension decreases (Uroz et al., 2018; Vianay et al., 2018; Nunes et al., 2020), likely reflecting adhesion complex disassembly (Dao et al., 2009), mediated by increased levels of Cyclin B1 (Gavet and Pines, 2010b; Jones et al., 2018). Overall, these observations highlight the interactions between physical forces and the cell cycle machinery and raise the interesting possibility that mechanical forces could directly influence the biochemical signals that control mitotic entry, contributing to the fidelity of chromosome segregation. As new tools emerge that allow us to probe the physical properties of cells, we will gain further insight on how the spatiotemporal dynamics of nuclear mechanics and nucleus-cytoskeleton coupling contribute to spindle assembly efficiency and chromosome segregation fidelity.

## Author Contributions

MD, JL, and JF jointly wrote the manuscript. JF provided the conceptual framework. All authors contributed to the article and approved the submission.

## Conflict of Interest

The authors declare that the research was conducted in the absence of any commercial or financial relationships that could be construed as a potential conflict of interest.
